# Reciprocity in Licensure

**Published:** 1905-05

**Authors:** 


### Reciprocity in Licensure.

THE subject of interstate endorsement of licenses to practise medicine has been discussed in these columns at appropriate intervals for years. Other journals have taken the matter up from time to time, correspondents have presented views, and the profession in general has become more or less satiated with the discussions in societies and in print. We have been silent on the question of late, because we have not observed any new thoughts of value or that offered any satisfactory solution of the problem.

The general belief among state medical examiners who have studied the question with care, is that uniformity in medical standards of education must obtain before there can be any substantial expectation of reciprocity. The action of the Medical College Association at Chicago a fortnight or more ago will have a tendency to expedite the reciprocal indorsement of licenses more than all the propositions that have been made in the journals in five years,—more than all the organisations of "reciprocating" boards.

Our attention has been drawn to this subject anew by the appearance of an editorial article in the *Fort Wayne Medical Journal-Magazine* for April, 1905, signed "M. F. P." This writer has stated the whole case so succinctly, so clearly and, withal, so conclusively that we take pleasure in reprinting his article, commending it to all interested:

### RECIPROCITY.

To give a dollar for a doughnut is not reciprocity in the true sense. It may suit the man who gives the doughnut for the dollar but not the one who gives the dollar for the doughnut. The former may be quite willing to continue reciprocating in this fashion indefinitely, but the latter will soon grow tired. States which admit to their examinations only those who give evidence of having taken a thorough medical course in a first-class college after having had an adequate preliminary education, cannot be expected to reciprocate with states whose requirements are much below these. One of the first moves in the direction of medical reciprocity between the different states should be an agreement between them on a common standard of requirements. One might, at first thought, conclude that any state should admit to practise within its boundaries any one licensed by another state whose requirements were more rigid than its own, and certainly admissions under such circumstances would prove beneficial rather than otherwise to the people of the state thus admitting them, but the writer is inclined to the opinion that we are still too human to thus tacitly acknowledge that our neighbors are better than we.

As against the plea for a common standard of requirements between the states it has been urged that it is not reasonable to require as much of him who desires to practise—say in Arkansas —as of him who desires to practise in New York. This argument is to the writer lame. If good, and followed to its logical conclusion, there should be several grades of requirements in each state, dependent upon the ability of the people of the community in which the applicant desires to practise, to pay for and to appreciate medical service. Next to "getting together," in the matter of requirements it should be kept constantly in mind that progress along this and all other lines of like legislation, will be rapid in proportion as we steer clear of partisan politics.

